# A visual and narrative timeline of US FDA milestones for Transcranial Magnetic Stimulation (TMS) devices

**DOI:** 10.1016/j.brs.2021.11.010

**Published:** 2021-11-11

**Authors:** Samantha L. Cohen, Marom Bikson, Bashar W. Badran, Mark S. George

**Affiliations:** Department of Biomedical Engineering, The City College of New York, CUNY, New York, NY, 10031, USA; Department of Biomedical Engineering, University of Southern California, Los Angeles, CA, 90007, USA; Department of Biomedical Engineering, The City College of New York, CUNY, New York, NY, 10031, USA; Department of Psychiatry, Medical University of South Carolina, Charleston, SC, 29425, USA; Department of Psychiatry, Medical University of South Carolina, Charleston, SC, 29425, USA; Ralph H. Johnson VA Medical Center, Charleston, SC, 29401, USA

Dear Editor,

It has been over a decade since the initial US Food and Drug Administration (FDA) approval of Transcranial Magnetic Stimulation (TMS). The technology was first approved for treating Major Depressive Disorder (MDD) in adults who have not responded satisfactorily to prior antidepressant medications in 2008 using the Neuronetics Neurostar System (DEN070003). Since then, refinement and optimization of TMS has paved the way to new and emerging technology that improves and broadens the clinical utility of TMS – such as pulse train protocols (e.g., iTBS and 18/20 Hz stimulation), neuronavigational systems, and electromagnetic coil technology. Alongside ongoing clinical trials, the approval of TMS therapies by the FDA has underpinned clinical adoption in the US. Here we summarize FDA regulatory milestones for TMS and provide a visual timeline of these approvals in Fig. 1.

Following the 2008 FDA approval of TMS for depression, the Nexstim eXimia NBS System was the first device FDA-cleared for cortical mapping (K091457) under the 510(k) pathway in 2009. Following this clearance, the Nexstim NBS System 4 was subsequently FDA-cleared for cortical mapping in 2012 with the addition of NEXSPEECH for localization and assessment of cortical areas of speech for pre-procedural planning (K112881).

The BrainsWay Deep TMS was cleared in 2013 for the treatment of depressive episodes in patients suffering from MDD who have failed to achieve sufficient improvement from prior antidepressant medication (K122288). This same year the eNeura Cerena System become the first cleared for acute treatment of pain associated with migraine with aura (K130556), using a handheld and delivered with minimal training. The following year, in 2014, eNeura was cleared for a portable device for treatment of migraine headache with aura with Spring TMS (K140094).

Moving into 2015, the field saw an expansion of two additional TMS devices which received clearance for the treatment of MDD in patients who have failed to receive improvements from prior antidepressant medication: Tonica Elektronik (Magventure)’s MagVita TMS (K150641) and Magstim’s Rapid^2^ System (K143531). Tele-EMG’s Neurosoft TMS (K160309) received clearance for the same indication a year later.

Aside from MDD, the next psychiatric condition that was FDA-approved was obsessive compulsive disorder (OCD). With the de novo pathway, BrainsWay’s Deep TMS System became the first to receive FDA approval as an adjunct for the treatment of OCD in adults in 2017 (DEN170078). The Nexstim Navigated Brain Therapy System 2 (K171902) was cleared in the same year for treatment of MDD in those who have failed to achieve satisfactory improvement from prior antidepressant medication in the current episode. Also cleared in 2017 by FDA regulation was the eNeura Spring TMS (K162797) for both acute and prophylactic treatment of migraine headache.

New pulse parameters, such as theta burst stimulation have also demonstrated utility in improving TMS therapeutic effects. The first device receiving clearance for the intermittent Theta Burst Stimulation (iTBS) protocol was the Tonika Elektronik (Magventure) Mag Vita TMS Therapy System with Theta Burst Stimulation (K173620) in 2018 for the treatment of MDD in adults previously failing to improve symptoms with antidepressant medication. Mag & More also received clearance for the Apollo TMS System (K180313) for the same indication that year.

The following year, two more devices received clearance for the iTBS protocol. Magstim’s Horizon TMS Therapy System with Navigation (K183376) for MDD added StimGuide for coil positioning using scalp landmarks, in addition to the iTBS protocol. Nexstim’s NBT System 2 (K182700) for MDD also added iTBS treatment capabilities. Also in 2019, the eNeura Spring TMS (K182976) expanded its indications for acute and prophylactic treatment of migraine headaches in adolescents and adults. The same year, Axilum Robotics’ TMS-Cobot TS MV (K182768) indicated for spatial positioning and orientation of the treatment coil of the MagVita TMS System received clearance. The de novo request by Neuronix for the neuroAD Therapy System (DEN160053) for the treatment of Alzheimer’s Disease was denied in 2019.

Neuronetics added the iTBS protocol with Neurostar Advanced Therapy with NeuroBurst (K201158) for the treatment of MDD in 2020. Tonica Elektronik also received clearance for the MagVenture TMS Therapy (K193006) for adjunctive treatment of OCD the same year. The Soterix Medical Neural Navigator (K191422) received clearance in 2020 as a neuronavigation system guided by MRI-based measurements for accurate positioning of the treatment coils. BrainsWay’s Deep TMS System (K200957) became the first to be cleared as an aid in short-term smoking cessation for adults in 2020. Finally, the clearance of BrainsWay’s Deep TMS System expanded the technology’s indications to include the treatment of depressive episodes and decreasing anxiety symptoms for those who may exhibit comorbid anxiety, previously failing to achieve satisfactory improvements with antidepressant medication for MDD (K210201).

The TMS field has grown substantially over the past two decades, moving from promising research findings to numerous FDA-approved medical devices with broad ranging treatment utility in neurological and psychiatric disorders. Several new indications such as OCD, anxiety comorbid with MDD, and smoking have emerged. Based on this record and ongoing efforts to enhance TMS technology and explore new treatment indications, further meaningful advances are expected.

## Figures and Tables

**Fig. 1.**
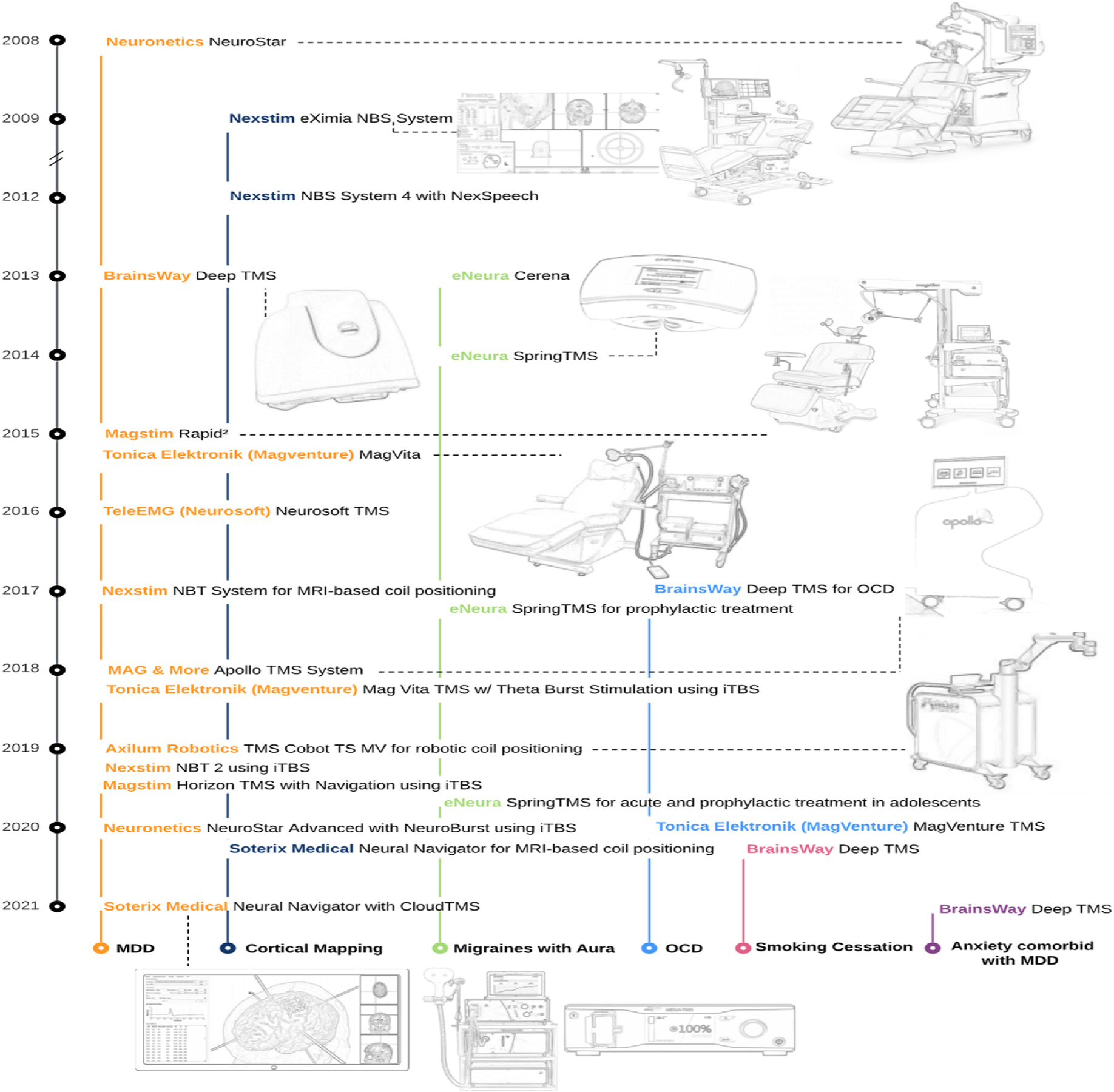
Timeline of US FDA milestones for Transcranial Magnetic Stimulation (TMS) devices.

